# A Genome-Wide Association Study of Prediabetes Status Change

**DOI:** 10.3389/fendo.2022.881633

**Published:** 2022-06-13

**Authors:** Tingting Liu, Hongjin Li, Yvette P. Conley, Brian A. Primack, Jing Wang, Wen-Juo Lo, Changwei Li

**Affiliations:** ^1^ College of Nursing, Florida State University, Tallahassee, FL, United States; ^2^ College of Nursing, University of Illinois at Chicago, Chicago, IL, United States; ^3^ School of Nursing, University of Pittsburgh, Pittsburgh, PA, United States; ^4^ College of Education and Health Professions, University of Arkansas, Fayetteville, AR, United States; ^5^ Department of Epidemiology, Tulane University School of Tropical Medicine and Public Health, New Orleans, LA, United States

**Keywords:** prediabetes status change, genome-wide association study, diabetes mellitus, type 2, normoglycemia

## Abstract

We conducted the first genome-wide association study of prediabetes status change (to diabetes or normal glycaemia) among 900 White participants of the Atherosclerosis Risk in Communities (ARIC) study. Single nucleotide polymorphism (SNP)-based analysis was performed by logistic regression models, controlling for age, gender, body mass index, and the first 3 genetic principal components. Gene-based analysis was conducted by combining SNP-based p values using effective Chi-square test method. Promising SNPs (p < 1×10-5) and genes (p < 1×10-4) were further evaluated for replication among 514 White participants of the Framingham Heart Study (FHS). To accommodate familial correlations, generalized estimation equation models were applied for SNP-based analyses in the FHS. Analysis results across ARIC and FHS were combined using inverse-variance-weighted meta-analysis method for SNPs and Fisher’s method for genes. We robustly identified 5 novel genes that are associated with prediabetes status change using gene-based analyses, including *SGCZ* (ARIC p = 9.93×10-6, FHS p = 2.00×10-3, Meta p = 3.72×10-7) at 8p22, *HPSE2* (ARIC p = 8.26×10-19, FHS p = 5.85×10-3, Meta p < 8.26×10-19) at 10q24.2, *ADGRA1* (ARIC p = 1.34×10-5, FHS p = 1.13×10-3, Meta p = 2.88×10-7) at 10q26.3, *GLB1L3* (ARIC p = 3.71×10-6, FHS p = 4.51×10-3, Meta p = 3.16×10-7) at 11q25, and *PCSK6* (ARIC p = 6.51×10-6, FHS p = 1.10×10-2, Meta p = 1.25×10-6) at 15q26.3. eQTL analysis indicated that these genes were highly expressed in tissues related to diabetes development. However, we were not able to identify any novel locus in single SNP-based analysis. Future large scale genomic studies of prediabetes status change are warranted.

## 1 Introduction

Diabetes is a major global public health challenge due to its high prevalence and associated morbidities and mortality (1). Prediabetes is a serious health condition where blood glucose levels are higher than normal, but not high enough to be diagnosed as type 2 diabetes (2). Approximately 88 million American adults aged 18 years or older, or more than 1 in 3 Americans, are estimated to have prediabetes (2). As the overweight and obesity rates continue to rise, these figures are expected to increase as well (3). To date, diabetes has emerged as a leading cause of blindness and end-stage renal failure and the seventh cause of mortality in the United States (2). The disease burden resulting from diabetes translates into a substantial economic toll. For example, the estimated total direct and indirect costs of diagnosed diabetes in the United States was $327 billion in 2017 (4).

Among people with prediabetes, about 5-10% will progress to overt diabetes annually, and a similar proportion will be converted back to normal (5). Prediabetes is also a critical time window for lifestyle interventions. Several landmark diabetes prevention clinical trials have provided robust evidence that participation in structured lifestyle interventions, focused on increased physical activity (2.5 to 4 hours/week), dietary modification (increased intake of whole grains, fiber, vegetables, and fruits; reduced intake of total and saturated fat, sugar, and refined grains), as well as weight reduction, improves blood glucose control and reduces more than 50% risk of diabetes (6–8).

Diabetes is a highly inheritable trait (9). Current genomic studies have identified many loci for diabetes and explained about 10% of the heritability (10). A large proportion of the heritability is still missing and many genes for diabetes are yet to be identified. Previous genomic studies of diabetes have primarily focused on the incidence of diabetes among population-based cohorts or have compared diabetes cases with controls in case-control studies (10–14). Several studies investigated genomic loci for diabetes phenotypes among participants with prediabetes (15, 16). None of those studies have investigated the prediabetes status change. Such investigation may help to identify novel genes for prediabetes status change. Therefore, the purpose of the current study was to identify genetic variants/genes associated with prediabetes status change by conducting genome-wide single nucleotide polymorphism (SNP)-based and gene-based association analyses among prediabetes participants of the Atherosclerosis Risk in Communities Study (ARIC).

## 2 Materials and Methods

### 2.1 Study Population

The ARIC is a population-based epidemiologic study among a total of 15,792 Black and White participants recruited from 4 communities (17). ARIC participants received extensive medical examinations every three years since the baseline in 1987-89 (17). The fourth follow-up visit was conducted in 1996-98 (18). ARIC data on genotypes, diabetes related measures, and important covariates were cataloged on the database of genotype and phenotype (dbGaP). We’ve received approval to use the data from both the Institute Review Board (IRB) at Tulane University and the dbGaP.

**Figure 1:**
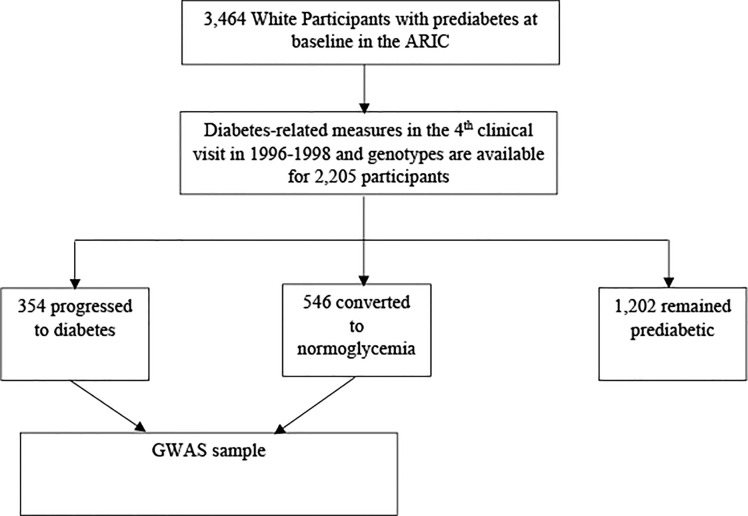
Flow Chart of Participant Selection in ARIC Cohort Study.

Due to very few Black participants with prediabetes in the ARIC study, our analysis was only conducted among White participants. As shown in the flow chart in **Figure 1**, a total of 3,464 White participants had prediabetes at baseline, diabetes related data was measured for 2,497 at the fourth clinical visit in 1996-98, and genome-wide genotypes were available for 2,205 of the participants on dbGaP. Among the 2,205 participants, 354 progressed to diabetes, 546 reversed to normal glycaemia, and 1,202 remained prediabetic. Our discovery stage analysis was conducted by comparing the 354 individual who progressed diabetes with the 546 participants who reversed to normal glycemia.

### 2.2 Genotyping, Quality Control, and Genotype Imputation

Genome-wide autosomal SNPs were genotyped using the Affymetrix 6.0 platform for a total of 8,620 unrelated White ARIC participants, and are available on dbGaP. Related pairs, duplicates, or gender misclassification were already evaluated and quality controlled in the genomic data. We performed further quality control and removed SNPs with Hardy-Weinberg equilibrium p < 1×10^-6^, missing rate>10%, or minor allele frequency (MAF) < 1% before genotype imputation. Individuals with missing genotype rate > 80% were also removed. After quality control, a total of 703,117 SNPs remained for genotype imputation. Imputation from the ALL ancestry panel of the 1000 Genome Phase III integrate Release Version 5 (19) was conducted for all White ARIC participants using MiniMac software (20). After imputation, SNPs with r^2^ < 0.30, MAF < 1%, or Hardy-Weinberg equilibrium p < 1×10^-6^ were removed, and a total of 10,008,913 SNPs, with fractional values ranging from 0 to 2, were retrieved for the 900 participants with prediabetes with changes in status for analysis.

### 2.3 Measurement of Prediabetes, Diabetes, and Covariates

In the ARIC, data on fasting blood glucose and diabetes medication use was collected at both baseline and the fourth clinical visit. This information was used to identify participants with prediabetes at baseline and to evaluate prediabetes status change in the fourth clinical visit according to the diagnosis guideline of the American Diabetes Association (21). Prediabetes was defined as fasting glucose level between 100 and 126 mg/dl and not taking glucose lowering medications. Diabetes was defined as fasting glucose ≥ 126 mg/dl, taking glucose lowering medications, or random glucose level ≥ 200 mg/dl. Those who had fasting or non-fasting glucose level <100 mg/dl and were not taking diabetes medication were defined as normal glycaemia.

Covariates included age, sex, body mass index (BMI), and the first 3 genetic principal components in the European-American sample. Age and sex were determined by self-report. Sex identity was checked by examining both X chromosome heterozygosity and the means of the intensive of SNP probes on the X and Y chromosomes (22). Population structure was investigated using principal components as described by Patterson et al. (23), and was available on the dbGaP. We adjusted for the first 3 genetic principal components to control for population substructure. Body weight and height were measured with participants wearing scrub suits and no shoes (24). Baseline BMI calculated as kg/m^2^ was included as a covariate.

### 2.4 Replication Study

**Figure 2:**
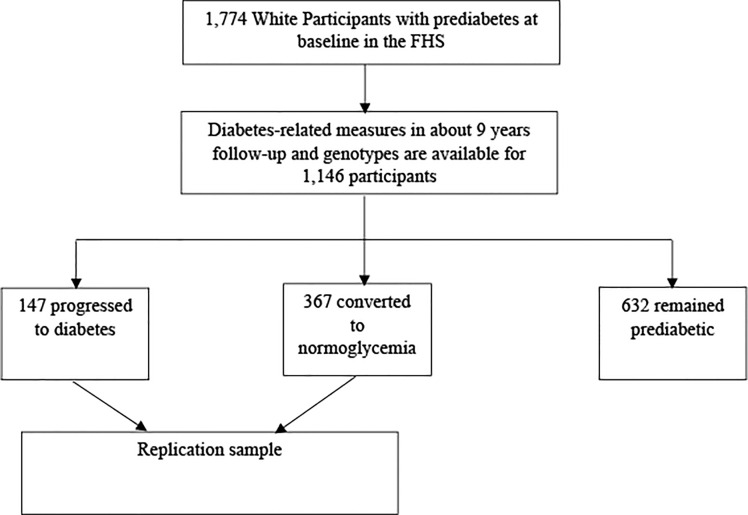
Flow Chart of Participant Selection in FHS Cohort Study.

We attempted to replicate promising ARIC findings among participants of the Framingham Heart Study (FHS). In FHS, fasting glucose was measured in visits 7, 8, 9, 10, and 13-23 in the original Framingham cohort. To be compatible with the ARIC study in length of follow-up time (≈ 9 years) and maximize study sample size, we treated visit 8 in 1962-66 as baseline and visit 13 in 1972-76 as the end follow-up period. In the offspring cohort, visit 1 in 1971-75 was selected as baseline and visit 2 in 1979-83 as end follow-up period. In the third-generation cohort, baseline was visit 1 in 2002-05 and end follow-up period was visit 2 in 2008-11. As shown in **Figure 2**, a total of 1,774 White participants with prediabetes were selected from the three Framingham cohorts, and 1,319 had genotypes available on the dbGaP. Diabetes status was determined cumulatively, and was available among a total of 1,146 participants with genotypes. In about 9 years’ follow-up, 147 participants developed diabetes, 367 participants reversed to normal, and 632 participants remained to be prediabetic. The replication analysis was performed by comparing the 147 individuals who progressed to diabetes with the 367 participants reversed to normal glycemia.

Genome-wide SNPs were genotyped using Affymetrix and Illumina platforms in FHS. The 1000 Genome genotype data for FHS was already imputed and cataloged on the dbGaP. According to the document of the FHS (25), before imputation, quality control removed SNPs with Hardy-Weinberg equilibrium p < 1×10^-6^, missing rate > 3.1%, MAF < 1%, missing physical position or not mapped to build 37 positions, Mendelian errors > 1000, or duplicate SNPs. MACH software was used for genotype phasing, followed by imputation using Minimac software (19, 20). SNPs within 3-Mb regions surrounding identified SNPs or SNPs in a promising gene in ARIC were imputed based on the ALL ancestry panel from the 1000G Phase I Integrated Release Version 3 Haplotypes (19, 20). After imputation, SNPs with r^2^ < 0.30, MAF < 1%, or Hardy-Weinberg equilibrium p < 1×10^-6^ were removed.

### 2.5 Statistical Analysis

The current analysis focuses on the comparison between prediabetes participants progressed to diabetes vs. those reversed to normal glycemia. We conducted both single SNP-based analysis and gene-based analysis as follows:

#### 2.5.1 Single Marker-Based Analysis

Logistic regression models were used to examine SNP-prediabetes status change associations (diabetes vs. normal glycaemia), after controlling for age, sex, body mass index, and the first 3 genetic principal components for population substructures in the ARIC. To accommodate familial relationships, generalized estimating equation models with compound symmetry correlation matrix were used to test SNP- prediabetes status change associations in the FHS, adjusting for the same covariates as in the ARIC. SNPs with discovery stage p < 1×10^-5^ in the ARIC were further evaluated in the FHS. Results from the ARIC and FHS were combined using inverse-variance-weighted meta-analysis method implemented in METAL software (26). After ensuring that the effect directions were consistent, SNPs with replication stage p < 0.05, and meta-analysis p < 5×10^-8^ were considered significant.

#### 2.5.2 Gene-Based Analysis

Similar to previous gene-based studies (27–29), SNPs within the 5-kb flanking regions of a gene were first mapped to the gene according to physical position. SNPs within 5-kb flanking regions of 2 genes were assigned to both genes. P values from single marker analysis were used to generate gene-based P values using the effective Chi-square test (ECS) method implemented in KGG software (30, 31). The ECS method uses SNP p values and LD information from the 1000G reference population of European ancestry to generate gene-based p values (30, 31). This method is more powerful for genes harboring multiple dense independent risk variants compared to the commonly used gene-based association test using Simes procedure (GATES) method (30, 31). Similar to previous genome-wide gene-based studies (28, 29), genes with p < 1×10^-4^ in the discovery stage analysis in ARIC were further evaluated for replication among FHS participants. In the FHS, SNPs from promising genes were tested for associations with prediabetes status change using methods described in the above single marker based analysis, and p values of these SNPs were again used to generate gene-based p values using the ECS method (30, 31). Fisher’s method was applied to combine gene-based p values across the ARIC and FHS (32). Genes with replication stage p < 0.05 and combined p < 2.5×10^-6^ (correcting for 20,000 genes across the genome: 0.05/20,000 = 2.5×10^-6^) were considered significant.

For significant SNPs and/or genes, we plotted regional SNP association plots using the KGG software (30), and searched their expression profiles in the Genotype-Tissue Expression (GTEx) project (33). The GETx project tested cis-eQTLs for all SNPs within 1 Mb flanking regions of the transcriptional start site of each gene in each tissue, using linear regression after correction for known and inferred technical covariates (34). Gene-level expression values were quantile normalized. Permutation-adjusted p value was computed for the most significant SNP in a gene, and was used to represent gene specific significance level. This approach corrects for multiple SNPs per gene (34). The eGene is defined as a gene with at least one SNP in cis significant association with expression differences of that gene after false discovery rate correction (34).

## 3 Results

Characteristics of both ARIC and FHS participants are shown in **Table 1**. ARIC participants were older and less likely to be male, compared to FHS participants. Participants of both studies were, on average, overweight or obese.

**Table 1:** Baseline characteristics of the ARIC and FHS participants by follow-up diabetes status.

Variables	ARIC				FHS			
	Normal (n=653)	preDM (n=1425)	DM (n=420)	*P*	Normal (n=367)	preDM (n=632)	DM (n=147)	*P*
Age, y, mean (SD)	54.8 (5.6)	54.9 (5.6)	54.7 (5.4)	0.1285	47.0 (9.6)	47.6 (9.9)	50.7 (8.2)	0.0003
Male, %	44.3%	59.4%	55.2%	<0.0001	62.1%	71.2%	61.9%	0.0047
BMI, kg/m^2^, mean (SD)	26.5 (4.5)	28.0 (4.5)	30.4 (5.0)	<0.0001	28.1 (5.6)	28.9 (4.7)	31.7 (6.6)	<0.0001

BMI, body mass index; DM, diabetes mellitus; FHS, Framingham Heart Study; preDM, pre-diabetes; SD, standard deviation.

**Figure 3:**
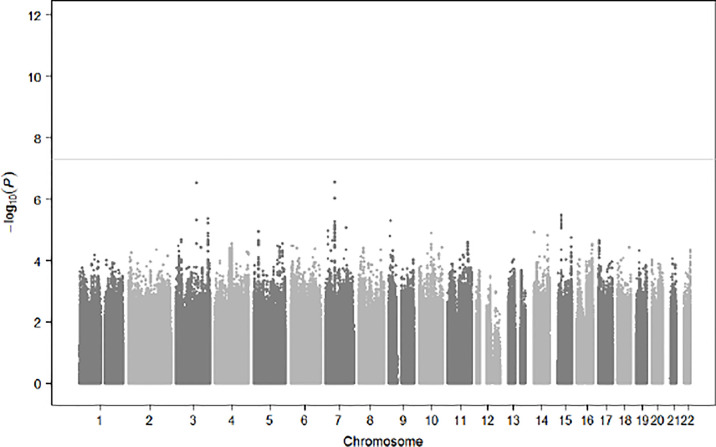
Manhattan Plot for Genome-Wide Single SNP-Based Analysis for Prediabetes Status Change.

**Figure 4:**
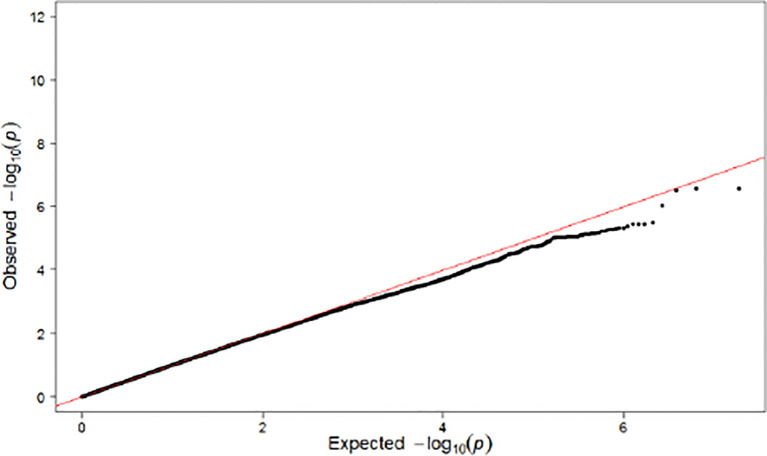
QQ Plot for Single SNP-Based Analysis Results.

Population substructures were well controlled (genomic inflation lambda = 1.027). Five independent loci (r^2^ < 0.3) reached suggestive significance (p < 1×10^-5^) in the discovery stage genome-wide analysis (**Figures 3**, **4**). As shown in **Table 2**, none of them were replicated in FHS. *GPR176* variant rs41497851 had a replication stage p = 0.0012. However, the direction of effect estimate was not consistent with that in the ARIC.

**Table 2:** Loci reaching suggestive significance level (P<1E-5) in ARIC.

rsID	Chr	Pos37	Nearest Gene	CA/AA	ARIC					FHS				
					CAF	r^2^	Beta	SE	*P*	CAF	r^2^	Beta	SE	*P*
rs36087584	3	119097548	ARHGAP31	C/T	0.39	0.80	0.67	0.13	2.99E-07	0.60	0.65	-0.07	0.18	0.6948
rs498414	3	185199267	MAP3K13	A/C	0.04	0.93	1.45	0.32	4.38E-06	0.97	0.51	0.63	0.52	0.2249
rs10229340	7	51596421	LOC105375277	G/A	0.48	0.74	-0.71	0.14	2.77E-07	0.53	0.59	-0.04	0.19	0.8368
rs4886290	13	61490922	LINC01442	T/C	0.11	0.99	0.80	0.18	7.53E-06	0.89	0.99	0.14	0.23	0.5465
rs41497851	15	40198302	GPR176	C/G	0.31	1.00	0.57	0.12	3.29E-06	0.71	1.00	-0.54	0.17	0.0012

AA, alternative allele; ARIC, Atherosclerosis Risk in Communities Study; CA, coded allele; CAF, coded allele frequency; FHS, Framingham Heart Study; SE, standard error; r^2^ is the imputation quality.

**Figure 5:**
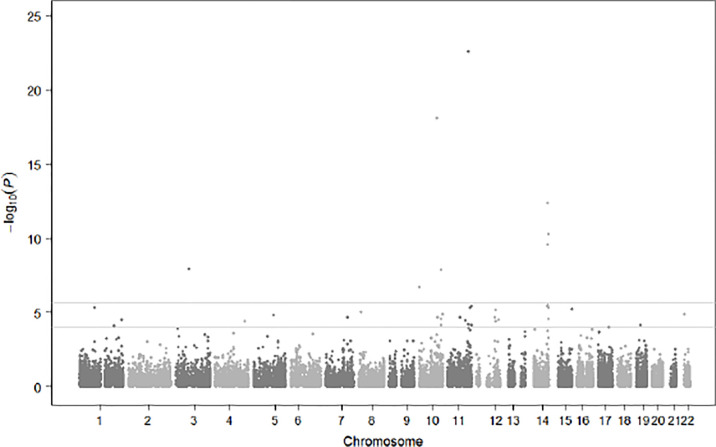
Manhattan Plot for Genome-Wide Gene-Based Analysis Results.

**Figure 6:**
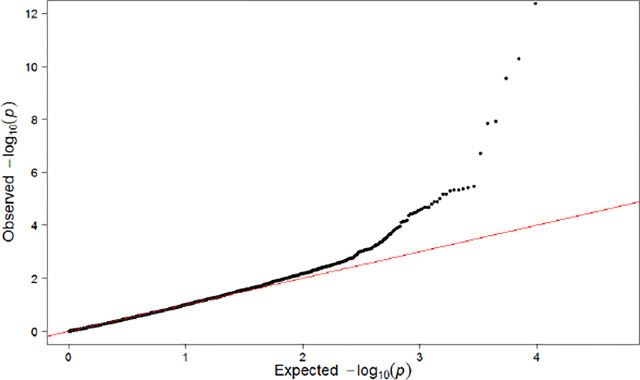
QQ Plot for Gene-Based Analysis Results.

**Figure 7:**
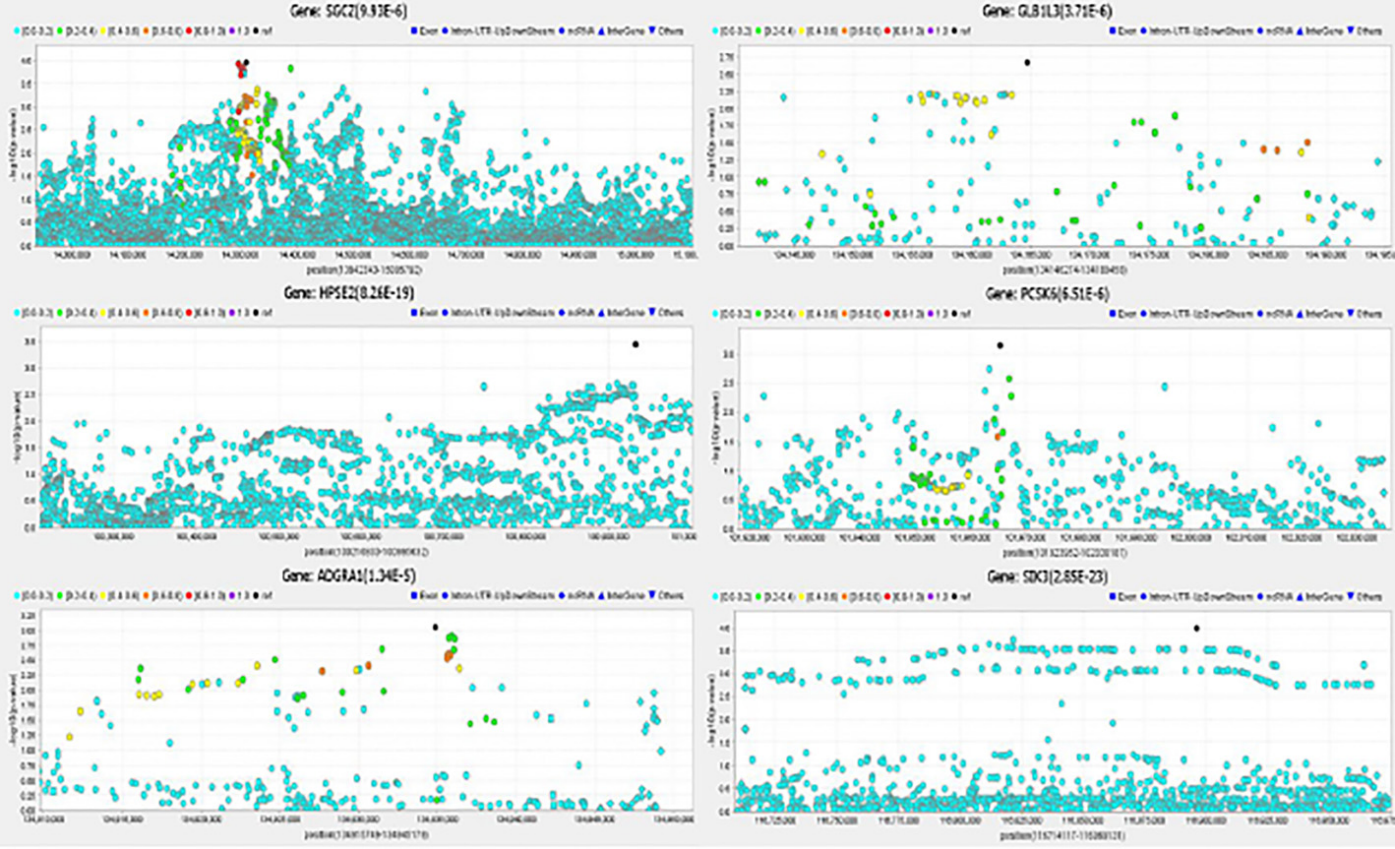
Regional Association Plots for Significant Genes.

A total of 36 genes located in 30 loci (within 1 mega base regions) had p < 1×10^-4^ in the discovery stage gene-based analysis (**Figures 5**, **6**), and were further evaluated for replication among FHS participants. Meta-analysis results for significant genes are shown in **Table 3**. Eight genes reached genome-wide significance in the discovery stage gene-based analysis, including *ZNF717* at (p = 1.20×10^-8^) 3p12.3, *DIP2C* (p = 1.99×10^-7^) at 10p15.3, *HPSE2* (p = 8.26×10^-19^) at 10q24.2, *UROS* (p = 1.39×10^-8^) at 10q26.2, *SIK3* (p = 2.85×10^-23^) at 11q23.3, *HHIPL1* (p = 4.27×10^-13^) at 14q23.2, *LINC00523* (p = 2.87×10^-10^) at 14q32.2, and *LOC102723354* (p = 5.34×10^-11^) at 14q32.33. The *HPSE2* was previously reported to be associated with type 1 diabetes (35) and the *LINC00523* was associated with type 2 diabetes (36). The combined analysis among ARIC and FHS identified five novel genes that reached genome-wide significance, including *SGCZ* (ARIC p = 9.93×10^-6^, FHS p = 2.00×10^-3^, Meta p = 3.72×10^-7^) at 8p22, *HPSE2* (ARIC p = 8.26×10^-19^, FHS p = 5.85×10^-3^, Meta p < 8.26×10^-19^) at 10q24.2, *ADGRA1* (ARIC p = 1.34×10^-5^, FHS p = 1.13×10^-3^, Meta p = 2.88×10^-7^) at 10q26.3, *GLB1L3* (ARIC p = 3.71×10^-6^, FHS p = 4.51×10^-3^, Meta p = 3.16×10^-7^) at 11q25, and *PCSK6* (ARIC p = 6.51×10^-6^, FHS p = 1.10×10^-2^, Meta p = 1.25×10^-6^) at 15q26.3. In addition, gene *SIK3* (ARIC p = 2.85×10^-23^) was marginally significant in FHS (p = 6.01×10^-2^), and reached genome-wide significance in the combined analysis (p < 1×10^-8^). Regional association plots for the 6 genes are demonstrated in **Figure 7**.

**Table 3:** Genes reached genome-wide significance in ARIC or meta-analysis.

Genes	Chr	Start Position (Build 37)	Function	ARIC *P*	FHS *P*	Meta *P*
***SGCZ***	8	13942343	PC	9.93E-06	2.00E-03	3.72E-07
*DIP2C*	10	320129	PC	1.99E-07	9.93E-01	3.25E-06
***HPSE2***	10	100216833	PC	8.26E-19	5.85E-03	<1.00E-23
*UROS*	10	127490625	PC	1.39E-08	8.85E-01	2.36E-07
***ADGRA1***	10	134915749	PC	1.34E-05	1.13E-03	2.88E-07
***SIK3***	11	116714117	PC	2.85E-23	6.01E-02	<1.00E-23
***GLB1L3***	11	134146274	PC	3.71E-06	4.51E-03	3.16E-07
*HHIPL1*	14	100111446	PC	4.27E-13	5.87E-01	7.52E-12
*LINC00523*	14	101123604	ncRNA	2.87E-10	5.82E-01	3.93E-09
*LOC102723354*	14	105560483	unknown	5.34E-11	1.75E-01	2.47E-10
***PCSK6***	15	101923952	PC	6.51E-06	1.10E-02	1.25E-06

Chr, chromosome; PC, protein coding; ncRNA, non-coding RNA; ARIC, Atherosclerosis Risk in Communities Study; FHS, Framingham Heart Study.

Bolded genes were successfully replicated in FHS and reached genome-wide significance level in the combined analyses.

eQTL analysis results are shown in **Table 4**. The 6 significant genes had eGenes in a variety of tissues, ranging from 2 tissues for the *SGCZ* gene to 14 tissues for the *HPSE2* gene. eGenes were identified for 5 genes in esophagus tissues (including mucosa and muscularis), 4 genes in tibial nerve tissue, 4 genes in adipose tissues (subcutaneous or visceral), 4 genes in aorta or coronary arteries, 4 genes in brain tissues, and 3 genes in thyroid tissue. eGenes were also identified for 2 genes in tissues of tibial artery, EBV-transformed lymphocytes, sigmoid colon, heart, lung, skeletal muscle, pituitary, stomach, vagina, testis, suprapubic skin, and lower leg skin, respectively. eGenes were identified in single tissue for 3 genes, including the *ADGRA1* in adrenal gland, liver, and spleen tissues, the *PCSK6* in atrial appendage, and pancreas tissues, and the *GLB1L3* in uterus.

**Table 4:** Tissues with eGenes for significant genes identified in genome-wide gene-based analyses.

Genes	NominalP-Value	Q-Value	Tissue
ADGRA1	6.17E-07	4.69E-03	Adrenal Gland
ADGRA1	8.22E-06	3.40E-02	Brain - Caudate (basal ganglia)
ADGRA1	2.78E-07	5.51E-03	Brain - Spinal cord (cervical c-1)
ADGRA1	4.26E-08	1.97E-04	Esophagus - Muscularis
ADGRA1	2.16E-06	1.86E-02	Liver
ADGRA1	4.91E-06	1.48E-02	Lung
ADGRA1	5.12E-07	1.46E-03	Nerve - Tibial
ADGRA1	4.76E-25	1.31E-18	Spleen
ADGRA1	1.63E-06	1.10E-02	Stomach
ADGRA1	2.02E-09	1.82E-05	Testis
ADGRA1	6.14E-11	5.59E-07	Thyroid
GLB1L3	1.51E-12	5.46E-08	Brain - Cerebellar Hemisphere
GLB1L3	8.10E-22	2.78E-16	Brain - Cerebellum
GLB1L3	9.36E-06	4.87E-02	Brain - Nucleus accumbens (basal ganglia)
GLB1L3	1.24E-09	1.99E-05	Colon - Sigmoid
GLB1L3	3.97E-12	5.52E-08	Esophagus - Muscularis
GLB1L3	6.85E-07	6.20E-03	Pituitary
GLB1L3	1.35E-06	5.72E-03	Skin - Not Sun Exposed (Suprapubic)
GLB1L3	2.26E-18	1.56E-13	Skin - Sun Exposed (Lower leg)
GLB1L3	9.80E-21	1.23E-15	Thyroid
GLB1L3	6.83E-07	1.15E-02	Uterus
GLB1L3	2.97E-06	3.97E-02	Vagina
HPSE2	7.99E-06	1.18E-02	Adipose - Subcutaneous
HPSE2	4.26E-44	6.30E-36	Artery - Aorta
HPSE2	3.16E-21	4.58E-15	Artery - Coronary
HPSE2	2.28E-51	1.32E-42	Artery - Tibial
HPSE2	1.27E-05	3.23E-02	Colon - Sigmoid
HPSE2	3.91E-05	4.17E-02	Esophagus - Mucosa
HPSE2	7.00E-09	3.69E-05	Esophagus - Muscularis
HPSE2	3.19E-20	4.24E-15	Lung
HPSE2	4.95E-05	4.14E-02	Nerve - Tibial
HPSE2	2.24E-06	5.45E-03	Skin - Not Sun Exposed (Suprapubic)
HPSE2	1.10E-11	1.02E-07	Skin - Sun Exposed (Lower leg)
HPSE2	4.96E-06	1.84E-02	Stomach
HPSE2	2.37E-10	1.15E-06	Thyroid
HPSE2	5.00E-06	3.49E-02	Vagina
PCSK6	7.28E-06	1.64E-02	Adipose - Subcutaneous
PCSK6	4.03E-09	4.14E-05	Adipose - Visceral (Omentum)
PCSK6	3.94E-07	2.10E-03	Artery - Aorta
PCSK6	8.07E-08	6.95E-04	Brain - Cerebellar Hemisphere
PCSK6	3.13E-07	4.28E-03	Cells - EBV-transformed lymphocytes
PCSK6	1.35E-07	6.31E-04	Esophagus - Mucosa
PCSK6	2.36E-07	1.06E-03	Esophagus - Muscularis
PCSK6	2.85E-12	6.72E-08	Heart - Atrial Appendage
PCSK6	1.46E-06	8.11E-03	Heart - Left Ventricle
PCSK6	2.95E-05	4.73E-02	Muscle - Skeletal
PCSK6	4.62E-09	2.53E-05	Nerve - Tibial
PCSK6	4.41E-07	3.10E-03	Pancreas
PCSK6	3.57E-06	1.86E-02	Pituitary
SGCZ	1.06E-05	3.10E-02	Adipose - Subcutaneous
SGCZ	7.00E-20	2.17E-14	Testis
SIK3	1.23E-06	6.02E-03	Artery - Aorta
SIK3	1.11E-05	2.35E-02	Artery - Tibial
SIK3	4.02E-06	4.28E-02	Brain - Hypothalamus
SIK3	1.01E-06	1.25E-02	Cells - EBV-transformed lymphocytes
SIK3	1.01E-10	1.19E-06	Esophagus - Mucosa
SIK3	3.10E-07	1.44E-03	Esophagus - Muscularis
SIK3	1.13E-08	1.15E-04	Heart - Left Ventricle
SIK3	7.82E-06	1.75E-02	Muscle - Skeletal
SIK3	1.74E-06	4.63E-03	Nerve - Tibial

eGene: defined as a gene with at least one SNP in cis significantly associated with expression differences of that gene after false discovery rate correction.

Q-value: p value after false-discovery rate correction.

## 4 Discussion

In the first genome-wide single SNP-based and gene-based analysis on prediabetes status change conducted in participants of European ancestry, we identified 5 novel genes, *SGCZ* at 8p22, *HPSE2* at 10q24.2, *ADGRA1* at 10q26.3, *GLB1L3* at 11q25, and *PCSK6* at 15q26.3 that were associated with prediabetes status change. In addition, gene-based analysis replicated a previously reported association between *LINC00523* gene and type 2 diabetes (36).

Gene *SGCZ* encoding zeta-sarcoglycan protein of the sarcoglycan complex (37) was associated with prediabetes status change in the current gene-based analysis. The *SGCZ* gene has been reported in genome-wide association studies (GWAS) of BMI (38) and obesity-related traits (39). These two phenotypes are highly associated with diabetes (40). In addition, another gene *SGCG* encoding protein in the sarcoglycan complex has been identified for diabetes among Punjabi Sikhs population in India (41). Animal studies have provided further evidence for the involvement of the *SGCZ* gene in diabetes development. For example, Groh and colleagues demonstrated that mice with sarcoglycan complex deficiency in adipose tissue and skeletal muscle had glucose-intolerance and insulin resistance specifically due to impaired insulin-stimulated glucose uptake in skeletal muscles (42). Despite biologically relevant in diabetes development, our study provided the first evidence that the *SGCZ* gene is involved in prediabetes status change in humans. Future large-scale genomic and functional studies of this gene are warranted to identify variants within this gene that are associated with prediabetes status change.

*HPSE2* gene encoding heparanase 2 (43), an enzyme that degrades heparin sulfate proteoglycans (44), was associated with prediabetes status change in the current study. The gene was suggested to be in association with type 1 diabetes in a previous GWAS meta-analysis (35). Furthermore, experimental studies indicated that the encoded heparanase was engaged in diabetes initiation and progression. Ziolkowski et al. reported that activation of heparanase was related to the destruction of pancreatic islets and inhibition of heparanase preserved intra-islet heparin sulfate, and protected mice from type 1 diabetes (45). In addition, degradation of heparin sulfate proteoglycans by heparanase creates a burst of cytokine release and can possibly promote beta cell death (46). In humans, studies demonstrated that serum and urinary heparanase levels were markedly elevated in type 2 diabetes patients compared to health controls (47) and were essential for the development of diabetic nephropathy (48). Our study provided robust evidence from population-based studies for the involvement of this gene in diabetes development. Further studies with a larger sample size and higher resolution genotypes are warranted to identify causal variants within this gene for prediabetes status change.

*ADGRA1* gene was associated with prediabetes status change in the current analysis. *ADGRA1* encodes adhesion G protein-coupled receptor A1 that belongs to the adhesion family of G-protein-coupled receptors (49). Receptors of this family regulate blood pressure (50), immune response (51), food intake (52) and development (53). These important functions are all related to glucose regulation. Therefore, G protein-coupled receptors have been new therapeutic targets for type 2 diabetes (54). Our study provided the first evidence that the *ADGRA1* gene is involved in prediabetes status change. Future studies are warranted to investigate the therapeutic role of this gene or its encoded protein in diabetes prevention among prediabetics.

*GLB1L3* gene encoding galactosidase beta 1 like 3 (55) was associated with prediabetes status change in the gene-based analysis. The biological relevance of this to glucose metabolism is not very clear. It may be involved in lactate production through converting serum lactose into glucose and galactose (56). Our study provided the first evidence of this gene in prediabetes status change in humans. Future works are warranted to delineate the causal role of this gene in blood glucose regulation.

Gene-based analysis also identified *PCSK6* gene that was associated with prediabetes status change. *PCSK6* gene encodes proprotein convertase subtilisin/kexin type 6, and plays important roles in the maturation of insulin receptor isoform B, and cholesterol and fatty acid metabolism (57, 58). In previous GWASs, *PCSK6* was reported to be associated with relative hand skill (59, 60). Large population based study indicated that left handedness increased risk of diabetes by 25% (61). Our finding may explain the mechanisms underlying the two observed associations. In addition, the *PCSK6* gene also activates corin, an important biomarker for salt-sensitive hypertension and diabetes (62, 63). More importantly, eQTL analysis identified that the *PCSK6* gene had SNPs in significant cis associations with its expression in the pancreas, pituitary, and omental adipose tissues. All these tissues are related to diabetes (64, 65). However, these are preliminary findings, and future functional study of the *PCSK6* gene in diabetes development are warranted.

*SIK3* gene was marginally significant in the replication stage analysis among FHS participants (p = 0.06), however, reached genome-wide significance in the combined analysis. This gene encoding SIK family kinase 3, is also biologically relevant to glucose metabolism, and is a potential target for diabetes therapeutics (66). SIK3 knocked-out mice had a high expression level of gluconeogenic gene, were leaner and more resistant to high-fat diet, and had excessive hypoglycemia (67). In humans, preliminary studies showed that SIK3 was downregulated in adipose tissues from obese or insulin-resistant individuals (68). Our study provided further evidence for the involvement of this gene in prediabetes status change. Future larger population-based studies are warranted to investigate the role of this gene in prediabetes status change.

eQTL analysis provided further evidence for the involvement of these 6 novel genes in diabetes development. Each of the novel genes had significant cis eQTL in tissues related to diabetes. For example, the *PCSK6* gene has significant eGene in the pancreas tissue. In addition, significant cis eQTLs were identified for more than 3 genes in tissues of esophagus, tibial nerve, adipose, aorta or coronary arteries, brain, and thyroid, respectively. Previous studies have shown that these tissues were all involved in diabetes pathogenesis. For example, diabetes may increase the risk of Barrett’s esophagus, indicating that genes involved in diabetes may contribute to Barrett’s esophagus pathogenesis (69). The cross-sectional area of the posterior tibial nerve was larger in patients with diabetes compared to healthy controls (70). Adipose tissue has long been a key target for diabetes pathophysiology and treatment (71). In addition, aortic, coronary, brain, and thyroid functions were also strongly associated with diabetes (72–74).

In comparison with previous GWAS meta-analysis of diabetes conducted in European population, the current study was able to identify several novel loci with a relatively small sample size. Such findings highlight the importance of examining novel disease phenotype (prediabetes status change) and conducting gene-based analysis to identify genomic mechanisms of diabetes risk. Furthermore, these findings contribute to understanding the mechanisms of diabetes development. Sequencing along with functional studies are needed to help delineate causal variants underlying the strong signals identified here. Finally, although it is not the aim of this current study, future research investigating the individual and overall contributions, such as polygenic risk scores, of previously reported genomic loci for diabetes phenotypes considering lifestyle behaviors and environmental exposures to the progression of prediabetes are warranted.

Our study represents the first GWAS of prediabetes status change conducted in participants of European ancestry. Additional study strengths included stringent quality control methods used in genotyping, genotype imputation, phenotypes, and measures of covariates for both the discovery and the replication stage samples. This can reduce errors in phenotype measures and increase statistical power in identifying both SNPs and genes underlying prediabetes status change. Furthermore, we used gene-based analysis to combine contributions of all variants in a gene. As noted above, this approach is more powerful than single SNP-based analysis (30, 31). More importantly, the longitudinal nature of the current study provided robust evidence that genetic factors play important roles in prediabetes status change. Findings from the current study may help to identify patients with prediabetes who were more likely to change their prediabetes status based on their genomic profiles and provided insight into the mechanisms underlying prediabetes status change.

There are also limitations for the study. First, due to limited sample size, we were not able to identify any single SNP associated with prediabetes status change. Future large-scale genomic studies among persons with prediabetes are warranted to robustly identify additional loci underlying prediabetes status change and to identify SNPs within genes reported in the current study. Second, In addition, the study findings may not be generalized to participants with prediabetes of other ancestry. Similar studies are needed to identify and map the relevant genes and SNPs involved in prediabetes status change in other populations. Third, the reason we compared participants who progressed to diabetes with those who reverted to normoglycemia mainly due to concerns of statistical power. We think if there are genetic variants involved in prediabetes status change, difference of the variants will be more prominent between the two groups, and therefore, we had higher statistical power to detect such variants. However, the genes for progression to diabetes and reversion to normoglycemia could be different, and these genes were not identified in this analysis, but is the goal of ongoing analyses. Finally, functions of the identified genes associated with prediabetes progression need to be investigated in cell lines and/or animal models to delineate their roles in diabetes development.

In conclusion, we conducted the first GWAS of prediabetes status change among participants of European ancestry using both single SNP-based and gene-based analyses. We robustly identified 5 novel genes associated with prediabetes status change through powerful gene-based analysis. The 5 genes are biologically relevant to diabetes and glucose regulation and warrant further investigations. Due to limited sample size, we were not able to identify any locus associated with prediabetes status change in the single SNP-based analysis. Future large-scale genomic studies among patients with prediabetes are warranted.

## Data Availability Statement

The data presented in the study are deposited in the database of Genotypes and Phenotypes (dbGaP) repository, accession number phs000090.v5.p1 for the ARIC study and phs000342.v20.p13 for the Framingham Heart Study.

## Ethics Statement

The studies involving human participants were reviewed and approved by Tulane University IRB. The ethics committee waived the requirement of written informed consent for participation.

## Author Contributions

TL, HL, and CL substantial contributed to conception and design. YC, BP, and JW contributed to acquisition of data. W-JL and CL contributed to analysis and interpretation of data. TL and HL contributed to draft the article. All authors contributed to the article and approved the submitted version.

## Funding

This study is funded by the National Institutes of Health (1P20GM109036-01A1) awarded to Changwei Li.

## Conflict of Interest

The authors declare that the research was conducted in the absence of any commercial or financial relationships that could be construed as a potential conflict of interest.

## Publisher’s Note

All claims expressed in this article are solely those of the authors and do not necessarily represent those of their affiliated organizations, or those of the publisher, the editors and the reviewers. Any product that may be evaluated in this article, or claim that may be made by its manufacturer, is not guaranteed or endorsed by the publisher.
